# A hypomorphic mutation in the mouse Csn1s1 gene generated by CRISPR/Cas9 pronuclear microinjection

**DOI:** 10.18699/VJ21.036

**Published:** 2021-05

**Authors:** A.V. Smirnov, T.А. Shnaider, A.N. Korablev, A.M. Yunusova, I.A. Serova, N.R. Battulin

**Affiliations:** Institute of Cytology and Genetics of the Siberian Branch of the Russian Academy of Sciences, Novosibirsk, Russia; Institute of Cytology and Genetics of the Siberian Branch of the Russian Academy of Sciences, Novosibirsk, Russia; Institute of Cytology and Genetics of the Siberian Branch of the Russian Academy of Sciences, Novosibirsk, Russia; Institute of Cytology and Genetics of the Siberian Branch of the Russian Academy of Sciences, Novosibirsk, Russia; Institute of Cytology and Genetics of the Siberian Branch of the Russian Academy of Sciences, Novosibirsk, Russia; Institute of Cytology and Genetics of the Siberian Branch of the Russian Academy of Sciences, Novosibirsk, Russia Novosibirsk State University, Novosibirsk, Russia

**Keywords:** сasein, CRISPR, pronuclear microinjection, hypomorphic mutation, казеин, CRISPR, пронуклеарная микроинъекция, гипоморфные мутации

## Abstract

Caseins are major milk proteins that have an evolutionarily conserved role in nutrition. Sequence variations in the casein genes affect milk composition in livestock species. Regulatory elements of the casein genes could be used to direct the expression of desired transgenes into the milk of transgenic animals. Dozens of casein alleles have been identified for goats, cows, sheep, camels and horses, and these sequence variants are associated with altered gene expression and milk protein content. Most of the known mutations affecting casein genes’ expression are located in the promoter and 3’-untranslated regions. We performed pronuclear microinjections with Cas9 mRNA and sgRNA against the first coding exon of the mouse Csn1s1 gene to introduce random mutations in the α-casein (Csn1s1) signal peptide sequence at the beginning of the mouse gene. Sanger sequencing of the founder mice identified 40 mutations. As expected, mutations clustered around the sgRNA cut site (3 bp from PAM). Most of the mutations represented small deletions (1–10 bp), but we detected several larger deletions as well (100–300 bp). Functionally most mutations led to gene knockout due to a frameshift or a start codon loss. Some of the mutations represented in-frame indels in the first coding exon. Of these, we describe a novel hypomorphic Csn1s1 (Csn1s1c.4-5insTCC) allele. We measured Csn1s1 protein levels and confirmed that the mutation has a negative effect on milk composition, which shows a 50 % reduction in gene expression and a 40–80 % decrease in Csn1s1 protein amount, compared to the wild-type allele. We assumed that mutation affected transcript stability or splicing by an unknown mechanism. This mutation can potentially serve as a genetic marker for low Csn1s1 expression.

## Abstract (ru)

Казеины – это основные молочные белки, которые играют важную эволюционно консервативную роль в питании. Вариации последовательности казеиновых генов влияют на состав молока у животных. Регуляторные элементы казеиновых генов можно использовать для управления экспрессией желаемых трансгенов в молоке трансгенных животных. Десятки аллелей казеина идентифицированы у коз, коров, овец, верблюдов и лошадей, и эти варианты связаны с измененной экспрессией генов и содержанием молочного белка. Большинство известных мутаций, влияющих на экспрессию генов казеина, находится в промоторных и 3’-нетранслируемых областях. Мы выполнили пронуклеарные микроинъекции с мРНК Cas9 и sgRNA против первого кодирующего экзона гена Csn1s1 мыши, чтобы ввести случайные мутации в последовательность сигнального пептида α-казеина (Csn1s1) в начале гена мыши. Секвенирование мышей-основателей по Сэнгеру выявило 40 мутаций. Как и ожидалось, мутации группировались вокруг сайта разреза sgRNA (3 п.н. от PAM). Большинство мутаций представляют собой небольшие делеции (1–10 п.н.), но мы также обнаружили несколько более крупных делеций (100–300 п.н.). Функционально большинство мутаций приводило к нокаутам генов из-за сдвига рамки считывания или потери стартовых кодонов. Некоторые из мутаций представлены инделами в рамке считывания в первом кодирующем экзоне. Из них мы описываем новый гипоморфный аллель Csn1s1 (Csn1s1c.4-5insTCC). Мы измерили уровни белка Csn1s1 и подтвердили, что мутация отрицательно влияет на состав молока, который показывает снижение экспрессии гена на 50 % и уменьшение количества белка Csn1s1 на 40–80 % по сравнению с аллелем дикого типа. Мы предположили, что мутация влияет на стабильность транскрипта или сплайсинг по неизвестному механизму. Эта мутация потенциально может служить генетическим маркером низкой экспрессии Csn1s1.

## Introduction

Caseins are major milk proteins that have an evolutionary conserved role in nutrition (Rijnkels et al., 2003). Casein locus has been studied for a long time to understand the principle of gene regulation and hormone-induced expression (Rijnkels et al., 2013; Dos Santos et al., 2015; Lee et al., 2017). At the same time, regulatory elements of the casein genes could be used to direct the expression of desired transgenes into milk of transgenic animals (Houdebine, 2009; Kim et al., 2015). This strategy is frequently employed to create “enriched” milk with improved composition (An et al., 2012; Wan et al., 2012; Yuan et al., 2014), or to achieve large scale production of recombinant human proteins in mouse models (Wu et al., 2012; Burkov et al., 2013; Qian et al., 2014) and in livestock species (Kalds et al., 2019). Milk-specific signal peptides are used in biotechnology to enhance recombinant protein secretion during lactation (Yu et al., 2006; Liu et al., 2014; Lu et al., 2019).

During breeding, casein genes acquired many sequence variations that can lead to altered gene expression and are characteristic of some goat and cow breeds (Yue et al., 2011; Fomichev et al., 2012; Guan et al., 2019). Many of these sequence variants could be used as markers for breeding. In dairy industry casein composition is an important milk trait directly influencing quality of dairy products (Sanchez et al., 2018; Cieslak et al., 2019). Hypomorphic casein mutation are also associated with other traits such as litter size (Wang et al., 2018). For example, K. Wang with colleagues showed that 11 bp del in the intron 8 of the goat Csn1s1 negatively affects the expression of the gene (Wang et al., 2018). Other known hypomorphic mutations are located in the promoter and 3′-untranslated regions (UTRs) of casein genes (Huang et al., 2012; Noce et al., 2016). 

Novel CRISPR methods greatly facilitate genome editing in farm animals (Kalds et al., 2019), including targeted transgene integration (Park et al., 2017) and mutation modeling (Li et al., 2017; Zhou et al., 2019). The latter approach has potential to explain the molecular mechanism of how hypomorphic mutations affect milk proteins. In this report, we used CRISPR/Cas9 to create a set of mutations within a signal peptide sequence of the α-casein (Csn1s1) gene in mice. One of the mutants was chosen to study effects of a small in-frame insertion on the Csn1s1 expression during lactation.

## Materials and methods

**Generation and genotyping of the Csn1s1 mutant mice.** In vitro transcription and purification of the gRNA were performed with MEGAshortscript™ T7 Transcription Kit (Thermo Fisher Scientific) and MEGAclear™ Transcription Clean-Up Kit (Thermo Fisher Scientific) according to the manufacturer’s protocol. Cas9 mRNA (GeneArt™ CRISPR Nuclease mRNA) was purchased from Thermo Fisher Scientific. 50 ng/μL Cas9 mRNA and 25 ng/μL gRNA (5′-GTGAG GATGAGGAGTTTCA-3′) were mixed in RNase-free water, backfilled into an injection needle with positive balancing pressure (Transjector 5246, Eppendorf) and injected into the cytoplasm of zygotes (C57BL/6 × CBA background). After injections, the embryos were cultured for 1 hour in drops of M16 medium at 37 °C and an atmosphere of 5 % CO2. The viable microinjected zygotes were transplanted the same day into oviducts of pseudopregnant CD-1 females (0.5 days after coitus). Isoflurane inhalation anesthesia was applied in these experiments.

Mutations were detected using PCR and Sanger sequencing of the target region of the Csn1s1 exon 2 (Supplementary 1)^1^. Primers for PCR were as follows: 5′-GCGCATAACTAAG CATCTTATGCT-3′ (forward primer), 5′-TGACTTGGAG TTTTAGATTTGGACA-3′ (reverse primer). Selected male mice founders were crossed with C57BL/6 females. For mutation c.4-5insTCC described in this paper, founder male was crossed with two F1 heterozygous daughters. Offspring was genotyped and two sibling females were selected for each group (wt, heterozygous or homozygous mutation) for further analysis.

Supplementary materials 1 and 2 are available at: http://www.bionet.nsc.ru/vogis/download/pict-2021-25/appx8.pdf


All experiments were conducted at the Centre for Genetic Resources of Laboratory Animals at the Institute of Cytology and Genetics, SB RAS (RFMEFI61914X0005 and RFMEFI61914X0010). All experiments were performed in accordance with protocols and guidelines approved by the Animal Care and Use Committee Federal Research Centre of the Institute of Cytology and Genetics, SB RAS operating under standards set by regulations documents Federal Health Ministry (2010/708n/RF), NRC and FELASA recommendations. Experimental protocols were approved by the Bioethics Review Committee of the Institute of Cytology and Genetics, SB RAS.

**Droplet digital PCR analysis.** Total cellular RNA was extracted from mouse mammary glands at day 8 of lactation using TRI Reagent (Sigma-Aldrich). 1 μg of total RNA was used to generate cDNA in a 20 μl reaction using RevertAid RT Kit (Thermo Fisher Scientific) with random hexamer primers according to the manufacturer’s instructions. Droplet Digital PCR (ddPCR) was performed using a QX100 system (Bio-Rad) with primers and probes specific for the Csn1s1 and Csn2 mouse transcripts (Supplementary 2). The primers and probes sequences were as follows: 5′-TGTAGTGGAT CAGGCACTGG-3′ (Csn1s1 forward primer), 5′-TCCTTG GAGACAATGGGCTT-3′ (Csn1s1 reverse primer), 5′-HEXCCAGTTCTCTGTTCAGCCCTTCCCACA-BHQ2–3′ (Csn1s1 probe), 5′-AGGACTTGACAGCCATGAAGG-3′ (Csn2 forward primer), 5′-ATGTTCAACAGATTCCTC ACTGGA-3′ (Csn2 reverse primer), 5′-FAM-ATCCTCGCC TGCCTTGTGGCCCTTGC-BHQ1–3′ (Csn2 probe). ddPCR reactions were set in 20 μl volumes containing 1× ddPCR™ Supermix for Probes (no dUTP), 900 nM primers and 250 nM probes, and 1 μl of 5000-fold diluted cDNA. ddPCR reactions for each sample were performed in duplicates. PCR was conducted according to the following program: 95 °C for 10 min, then 40 cycles of 95 °C for 30 s and 61 °C for 1 min, with a ramp rate of 2 °C per second, and a final step at 98 °C for 5 min. The results were analyzed using QuantaSoft software (Bio-Rad). Concentrations of cDNA copies of Csn1s1 and Csn2 were derived from ddPCR and relative expression of Csn1s1 to Csn2 was calculated for each animal.

**Milk and mammary gland protein analysis.** Milk was obtained from narcotized female mice at day 8 of lactation after oxytocin administration (Uusi-Oukari et al., 1997). The milk was collected with a pipette attached to an aspiration device, transferred into a microcentrifuge tube and stored at –80 °C. Inguinal mammary glands (MGs) were extracted from the same (euthanized) mice and stored at –80 °C. For protein extraction MGs were minced in Dounce homogenizers, resuspended in RIPA buffer (150 mM NaCl, 1 % Nonidet P-40, 0.5 % sodium deoxycholate, 0.1 % SDS, 15 mM Tris pH 7.4) with protease inhibitor cocktail (1x Complete ULTRA (Roche), 1x PhosSTOP (Roche), 5 mM NaF (Sigma)). The lysates were incubated on ice for 30 min and then centrifuged at 4 °C for 10 min at 10 000 g. Supernatant was sonicated and stored at –80 °C. Total protein concentrations for milk and MG lysates were determined with Pierce™ BCA Protein Assay Kit (Thermo Fisher Scientific), according to the manufacturer’s instructions. To prepare samples for SDSPAGE, milk or MG protein samples were mixed with RIPA and SDS-PAGE loading buffer (Bio-Rad) to a final concentration of 1 μg/μl and heated at 65 °C for 20 minutes. The samples (25 μg) were separated on a 12 % polyacrylamide gel and stained with Coomassie Blue G-250. ThermoFisher PageRuler™ Prestained Protein Ladder (10 to 180 kDa) was used as a protein molecular weight marker. Csn1s1, albumin and total protein concentrations were evaluated using Quantity One (Bio-Rad) and ImageJ software.

Coomassie-stained gel was used for wet transfer of the proteins to PVDF membrane (0.45 μm Immobilon-P, Merck). The membrane was blocked with 5 % milk in TBST (20 mM Tris pH 7.5, 150 mM NaCl, 0.1 % Tween 20) for 2 hours and incubated with primary mouse anti-Csn1s1 antibodies (1:1000) (sc-373711, Santa Cruz Biotechnology) overnight at 4 °C. Next day membrane was repeatedly washed with TBST and incubated with secondary mouse HRP-antibodies (1:1000) (sc-516102, Santa Cruz Biotechnology) at 25 °C for 2 hours. Immunodetection was performed with ECL substrate solution (Millipore Corporation, Billerica, MA, USA), according to the manufacturer’s instructions.

## Results

**Generation of the Csn1s1 mutant mice**

**Fig. 1.**
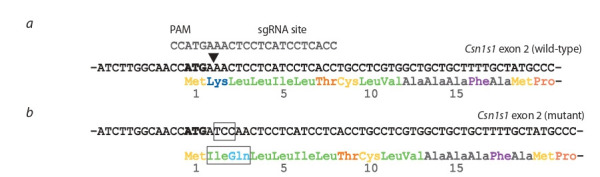
Generation of the Csn1s1 mutant mice by CRISPR/Cas9 pronuclear microinjection. a – sequence of the first coding exon (63 bp) of the Csn1s1 gene (exon 2, NM_007784.3). Black arrowhead indicates Cas9 cleavage site. Complementary 20 bp sgRNA sequence with PAM is shown above; b – sequence of the mutated Csn1s1 exon with a 3 bp insertion in the second codon (framed). Start codon (ATG) is highlighted.

We performed pronuclear microinjections with Cas9 mRNA and sgRNA against the first coding exon of the mouse Csn1s1 gene (Fig. 1, a). Cas9-induced mutations in this region could potentially affect signal peptide coding sequence and lead to altered milk composition. We screened founder mice by Sanger sequencing and identified multiple random mutations at the Cas9 cut site (presented in Supplementary 1). The sgRNA targeted the Csn1s1 site with high efficiency as we detected 41 mutant alleles, 4–5 mosaic alleles (additional background signal) and only 4–5 wild-type alleles in 20 founder mice (~90 % allele mutation efficiency). As expected, mutations clustered around the sgRNA cut site (3 bp from PAM). Most of the mutations represented small deletions (1–10 bp), but we detected several larger deletions as well (100–300 bp). Of note, some of the unique mutation variants had increased incidence rate. For example, 12 bp deletion (GAAACTCCTCAT) arose independently four times and another 10 bp deletion (CCATGAAACT) – three times (see Supplementary 1). We suspect this bias towards some variants is caused by microhomology-mediated end-joining (MMEJ), since these two mutations are flanked with 3 and 2 similar nucleotides (CAT and CC, respectively) (see Supplementary 1). Although mutations were mostly deleterious for the gene expression and led to a Csn1s1 knockout (KO) by frameshift, several in-frame mutation variants resulted in subtle changes in signal peptide coding sequence without KO (see Supplementary 1). We selected one of the mutants, tagged Csn1s1c.4-5insTCC, which had a 3 bp insertion following the start codon (see Fig. 1, b). To study gene expression and milk composition we chose 6 female siblings (2 wild-type, 2 heterozygotes, 2 homozygotes) from the Csn1s1c.4-5insTCC line for milk and mammary glands collection.

**Mutation c.4-5insTCC leads to reduced expression of the Csn1s1 gene**

**Fig. 2.**
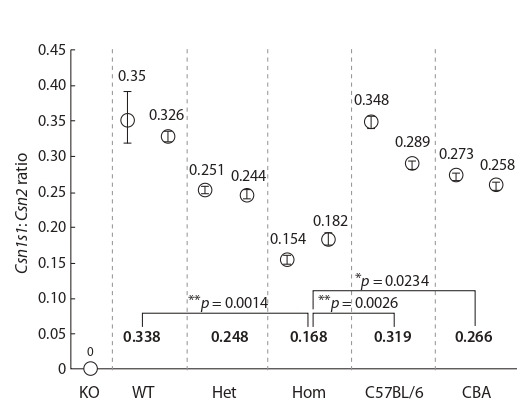
. Droplet digital PCR analysis of the Csn1s1 and Csn2 expression in mammary gland. Data presented as ratios of Csn1s1/Csn2 transcripts. Error bars – standard deviations. Bold numbers at the bottom represent mean ratios for two females of each group. KO – Csn1s1 knockout female; WT, Het, Hom – siblings with corresponding mutation genotype; C57BL/6, CBA – females from the inbred strains. Statistics: one-way ANOVA.

We estimated the Csn1s1 gene expression in mammary glands of wild-type and mutant mice at day 8 of lactation using droplet digital PCR (ddPCR). We selected Csn2 (β-casein) as a reference gene as it has a similar expression profile in mammary gland (see Supplementary 2). ddPCR analysis revealed that Csn1s1:Csn2 ratio was roughly 1:3 (0.338) in wild-type siblings (Fig. 2), which is in agreement with published data (Yamaji et al., 2013). Heterozygous and homozygous Csn1s1c.4-5insTCC siblings showed lower Csn1s1 expression with ratios around 1:4 (0.248) and 1:6 (0.168), respectively (see Fig. 2), compared to wild-type siblings. We also used females from the parental strains C57BL/6 and CBA as controls for normal caseins level (see Fig. 2). In rare cases, Cas9 activity can provoke rearrangements near the target locus.

We sequenced mutation-flanking regions, including the Csn1s1 promoter and surrounding introns (2.3 kb+1.1 kb) from homozygous mice (data not shown). We also sequenced top two off-targets for the Csn1s1 sgRNA in the F0 founder (Chr1:24388301; Chr4:140044179). No mutations were found in the examined sequences. Thus, we could confirm that the 3 bp in-frame insertion in the first coding exon led to a 30–50 % decrease in the Csn1s1 gene expression. 

**Milk protein composition in c.4-5insTCC mice**

**Fig. 3.**
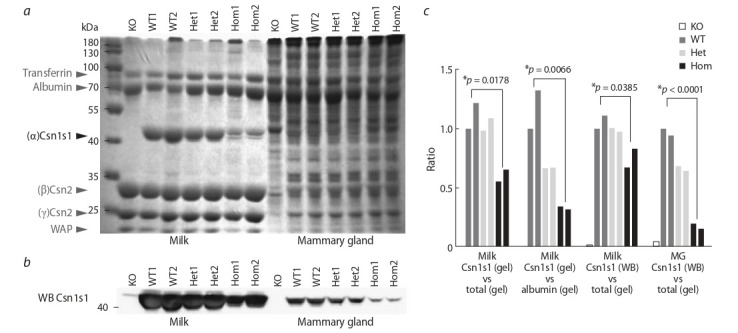
Detection of the Csn1s1 protein in milk and mammary glands of the mutant mice. a – coomassie-stained 12 % SDS-polyacrylamide gel analysis of the whole milk and mammary gland lysates from wild-type (WT1, WT2), heterozygous (Het1, Het2) and homozygous (Hom1, Hom2) Csn1s1 mutant mice. KO – the Csn1s1 knockout mice. ThermoFisher PageRuler™ Prestained Protein Ladder (10 to 180 kDa) was used as a protein molecular weight marker. Major milk proteins are indicated with arrows. Csn1s1 protein expected size – 43 kDa; b – Western blot of the same Coomassie-stained gel transferred to a PVDF membrane; c – quantitation of Csn1s1 protein in the milk of mutant mice using data from Fig. 3, a and b. Intensity of the Csn1s1 protein band was calculated in relation to the whole milk protein signal (total protein). MG – mammary gland. ImageJ software was used for analysis. Statistics: one-way ANOVA, p-values shown for WT vs homozygotes comparisons. One of the WT controls (WT1) was set to 1 (100 %).

We measured Csn1s1 protein levels and confirmed that mutation has a negative effect on milk composition. We collected milk and mammary glands from lactating females at the day 8 of lactation. Csn1s1 knockout mouse was taken from another experiment (“KO”) as additional control for the Csn1s1 levels. Separation of milk proteins on 12 % SDS‐PAGE resulted in a typical band pattern for mouse milk (Fig. 3, a). In wildtype mice, Csn1s1 represents a major protein fraction and corresponds to approx. 30 % of total milk protein (Kolb et al., 2011). In homozygous mutants, loss of Csn1s1 could be observed both at the Coomassie-stained gel (see Fig. 3, a) and after western blotting (see Fig. 3, b). Csn1s1 levels fell down to 30 % in homozygotes, both for milk and for mammary gland lysates (see Fig. 3, c). However, exact ratios varied depending on the control protein band used for calculations. For instance, we performed the following calculations for the milk Csn1s1: Csn1s1 (gel) vs total protein (gel) – 40 % reduction; Csn1s1 (gel) vs albumin (gel) – 70 % reduction; Csn1s1 (western blot membrane) vs total protein (gel) – 80 % reduction. This effect was even more pronounced in mammary gland lysates (intracellular casein levels): Csn1s1 (western blot membrane) vs total gel – 80 % reduction.

## Discussion

We report a novel in-frame Csn1s1 hypomorphic mutation that leads to a 50 % gene expression decrease in mice. In most cases, mutation effect is tied to disruption of a regulatory element (enhancer, promoter, UTR, miRNA site) (Hogg, Harries, 2014). Frame-shifting indels in the coding sequence could initiate transcript surveillance pathway called nonsense-mediated mRNA decay (NMD) (Popp, Maquat, 2016; Lindeboom et al., 2019). Alternatively, in-frame mutations can lead to exon removal by alternative splicing (Mucaki et al., 2020; Thompson et al., 2020). Essentially, exon skipping could be promoted by internal exon splicing enhancers and suppressors (ESEs and ESSs) which are hard to predict (Sterne-Weiler, Sanford, 2014; Tuladhar et al., 2019), unlike typical splice site mutations (Cartegni et al., 2002). In our mutant mice, promoter had no alterations as the mutation happened in the coding sequence, quite far from a transcription start site. We assumed that it affected transcript stability or splicing by unknown mechanism. It should also be noted that mutated Csn1s1 protein was still secreted in milk, thus the function of N-terminal signal peptide was not critically affected by the mutation. 

## Conclusion

We demonstrated that CRISPR/Cas9 approach could be conveniently exploited to induce a spectrum of mutations in the Csn1s1 gene either by random mutagenesis, or, ideally, by a set of single-stranded oligo DNA nucleotides (ssODNs). Our results warn that careful examination of the gene’s expression is required in addition to protein analysis. 

## Conflict of interest

The authors declare no conflict of interest.

## References

An L.Y., Yuan Y.G., Yu B.L., Yang T.J., Cheng Y. Generation of human lactoferrin transgenic cloned goats using donor cells with dual
markers and a modified selection procedure. Theriogenology. 2012;
78:1303-1311. DOI 10.1016/j.theriogenology.2012.05.027.

Burkov I.A., Serova I.A., Battulin N.R., Smirnov A.V., Babkin I.V.,
Andreeva L.E., Dvoryanchikov G.A., Serov O.L. Expression of the
human granulocyte-macrophage colony stimulating factor (hGMCSF) gene under control of the 5′-regulatory sequence of the goat
alpha-S1-casein gene with and without a MAR element in transgenic
mice. Transgenic Res. 2013;22:949-964. DOI 10.1007/s11248-013-
9697-4.

Cartegni L., Chew S.L., Krainer A.R. Listening to silence and understanding nonsense: exonic mutations that affect splicing. Nat. Rev.
Genet. 2002;3(4):285-298. DOI 10.1038/nrg775. PMID 11967553.

Cieslak J., Wodas L., Borowska A., Pawlak P., Czyzak-Runowska G.,
Wojtowski J., Puppel K., Kuczynska B., Mackowski M. 5′-flanking
variants of equine casein genes (CSN1S1, CSN1S2, CSN2, CSN3)
and their relationship with gene expression and milk composition.
J. Appl. Genet. 2019;60(1):71-78. DOI 10.1007/s13353-018-0473-2.

Dos Santos C.O., Dolzhenko E., Hodges E., Smith A.D., Hannon G.J.
An epigenetic memory of pregnancy in the mouse mammary gland.
Cell Rep. 2015;11:1102-1109. DOI 10.1016/j.celrep.2015.04.015.

Fomichev K.A., Sazanova A.L., Malewski T., Kaminski S., Sazanov A.A. Associations between two novel rSNPs in 5′-flanking region of the bovine casein gene cluster and milk performance traits.
Gene. 2012;496:49-54. DOI 10.1016/j.gene.2011.12.038.

Guan D., Mármol-Sánchez E., Cardoso T.F., Such X., Landi V., Tawari N.R., Amills M. Genomic analysis of the origins of extant
casein variation in goats. J. Dairy Sci. 2019;102:5230-5241. DOI
10.3168/jds.2018-15281.

Hogg D.R., Harries L.W. Human genetic variation and its effect on
miRNA biogenesis, activity and function. Biochem. Soc. Trans. 2014;
42(4):1184-1189. DOI 10.1042/BST20140055. PMID 25110023.

Houdebine L.M. Production of pharmaceutical proteins by transgenic
animals. Comp. Immunol. Microbiol. Infect. Dis. 2009;32:107-121.
DOI 10.1016/j.cimid.2007.11.005.

Huang W., Peñagaricano F., Ahmad K.R., Lucey J.A., Weigel K.A.,
Khatib H. Association between milk protein gene variants and protein composition traits in dairy cattle. J. Dairy Sci. 2012;95:440-449.
DOI 10.3168/jds.2011-4757.

Kalds P., Zhou S., Cai B., Liu J., Wang Y., Petersen B., Sonstegard T.,
Wang X., Chen Y. Sheep and goat genome engineering: from random transgenesis to the CRISPR Era. Front. Genet. 2019;10:750.
DOI 10.3389/fgene.2019.00750. eCollection2019.

Kim J.J., Yu J., Bag J., Bakovic M., Cant J.P. Translation attenuation
via 3′ terminal codon usage in bovine csn1s2 is responsible for the
difference in αs2- and β-casein profile in milk. RNA Biol. 2015;12:
354-367. DOI 10.1080/15476286.2015.1017231.

Kolb A.F., Huber R.C., Lillico S.G., Carlisle A., Robinson C.J., Neil C.,
Petrie L., Sorensen D.B., Olsson I.A., Whitelaw C.B. Milk lacking
α-casein leads to permanent reduction in body size in mice. PLoS
One. 2011;6:e21775. DOI 10.1371/journal.pone.0021775.

Lee H.K., Willi M., Wang C., Yang C.M., Smith H.E., Liu C., Hennighausen L. Functional assessment of CTCF sites at cytokinesensing mammary enhancers using CRISPR/Cas9 gene editing in
mice. Nucleic Acids Res. 2017;45:4606-4618. DOI 10.1093/nar/
gkx185.

Li W.R., Liu C.X., Zhang X.M., Chen L., Peng X.R., He S.G., Lin J.P.,
Han B., Wang L.Q., Huang J.C., Liu M.J. CRISPR/Cas9-mediated
loss of FGF5 function increases wool staple length in sheep. FEBS J.
2017;284:2764-2773. DOI 10.1111/febs.14144.

Lindeboom R.G.H., Vermeulen M., Lehner B., Supek F. The impact
of nonsense-mediated mRNA decay on genetic disease, gene editing and cancer immunotherapy. Nat. Genet. 2019;51(11):1645-
1651. DOI 10.1038/s41588-019-0517-5. Epub 2019 Oct 28. PMID
31659324. PMCID PMC6858879.

Liu H.C., Pai S.Y., Chen H.L., Lai C.W., Tsai T.C., Cheng W.T.,
Yang S.H., Chen C.M. Recombinant Derp5 allergen with αS1-casein
signal peptide secreted in murine milk protects against dust mite
allergen-induced airway inflammation. J. Dairy Sci. 2014;97:6792-
6803. DOI 10.3168/jds.2014-8484.

Lu R., Zhang T., Song S., Zhou M., Jiang L., He Z., Yuan Y., Yuan T.,
Lu Y., Yan K., Cheng Y. Accurately cleavable goat β-lactoglobulin
signal peptide efficiently guided translation of a recombinant human
plasminogen activator in transgenic rabbit mammary gland. Biosci.
Rep. 2019;39:6. DOI 10.1042/BSR20190596.

Mucaki E.J., Shirley B.C., Rogan P.K. Expression changes confirm
genomic variants predicted to result in allele-specific, alternative
mRNA splicing. Front. Genet. 2020;11:109. DOI 10.3389/fgene.
2020.00109. PMID 32211018. PMCID PMC7066660.

Noce A., Pazzola M., Dettori M.L., Amills M., Castelló A., Cecchinato A., Bittante G., Vacca G.M. Variations at regulatory regions of
the milk protein genes are associated with milk traits and coagulation properties in the Sarda sheep. Anim. Genet. 2016;47:717-726.
DOI 10.1111/age.12474.

Park K.E., Powell A., Sandmaier S.E., Kim C.M., Mileham A., Donovan D.M., Telugu B.P. Targeted gene knock-in by CRISPR/Cas ribonucleoproteins in porcine zygotes. Sci. Rep. 2017;7:42458. DOI
10.1038/srep42458.

Popp M.W., Maquat L.E. Leveraging rules of nonsense-mediated
mRNA decay for genome engineering and personalized medicine.
Cell. 2016;165(6):1319-1322. DOI 10.1016/j.cell.2016.05.053.
PMID 27259145. PMCID PMC4924582.

Qian X., Kraft J., Ni Y., Zhao F.Q. Production of recombinant human
proinsulin in the milk of transgenic mice. Sci. Rep. 2014;4:6465.
DOI 10.1038/srep06465.

Rijnkels M., Elnitski L., Miller W., Rosen J.M. Multispecies comparative analysis of a mammalian-specific genomic domain encoding secretory proteins. Genomics. 2003;82:417-432. DOI 10.1016/s0888-
7543(03)00114-9.

Rijnkels M., Freeman-Zadrowski C., Hernandez J., Potluri V., Wang L.,
Li W., Lemay D.G. Epigenetic modifications unlock the milk protein gene loci during mouse mammary gland development and differentiation. PLoS One. 2013;8:e53270. DOI 10.1371/journal.pone.
0053270.

Sanchez M.P., Wolf V., El Jabri M., Beuvier E., Rolet-Répécaud O.,
Gaüzère Y., Minéry S., Brochard M., Michenet A., Taussat S., Barbat-Leterrier A., Delacroix-Buchet A., Laithier C., Fritz S., Boichard D. Short communication: Confirmation of candidate causative
variants on milk composition and cheesemaking properties in Montbéliarde cows. J. Dairy Sci. 2018;101:10076-10081. DOI 10.3168/
jds.2018-14986.

Sterne-Weiler T., Sanford J.R. Exon identity crisis: disease-causing mutations that disrupt the splicing code. Genome Biol. 2014;15(1):201.
DOI 10.1186/gb4150. PMID 24456648. PMCID PMC4053859.

Thompson B.A., Walters R., Parsons M.T., Dumenil T., Drost M.,
Tiersma Y., Lindor N.M., Tavtigian S.V., de Wind N., Spurdle A.B.,
InSiGHT Variant Interpretation Committee. Contribution of mRNA
splicing to mismatch repair gene sequence variant interpretation.
Front. Genet. 2020;11:798. DOI 10.3389/fgene.2020.00798. PMID
32849802. PMCID PMC7398121.

Tuladhar R., Yeu Y., Tyler Piazza J., Tan Z., Rene Clemenceau J., Wu X.,
Barrett Q., Herbert J., Mathews D.H., Kim J., Hyun Hwang T.,
Lum L. CRISPR-Cas9-based mutagenesis frequently provokes
on-target mRNA misregulation. Nat. Commun. 2019;10(1):4056.
DOI 10.1038/s41467-019-12028-5. PMID 31492834. PMCID
PMC6731291.

Uusi-Oukari M., Hyttinen J.M., Korhonen V.P., Västi A., Alhonen L.,
Jänne O.A., Jänne J. Bovine alpha s1-casein gene sequences direct
high level expression of human granulocyte-macrophage colonystimulating factor in the milk of transgenic mice. Transgenic Res.
1997;6:75-84. DOI 10.1023/a:1018461201385.

Wan Y.J., Zhang Y.L., Zhou Z.R., Jia R.X., Li M., Song H., Wang H.Z.,
Wang L.Z., Zhang G.M., You J.H., Wang F. Efficiency of donor cell
preparation and recipient oocyte source for production of transgenic
cloned dairy goats harboring human lactoferrin. Theriogenology.
2012;78:583-592. DOI 10.1016/j.theriogenology.2012.03.004.

Wang K., Yan H., Xu H., Yang Q., Zhang S., Pan C., Chen H., Zhu H.,
Liu J., Qu L., Lan X. A novel indel within goat casein alpha S1 gene
is significantly associated with litter size. Gene. 2018;671:161-169.
DOI 10.1016/j.gene.2018.05.119.

Wu X., Lin Y., Xiong F., Zhou Y., Yu F., Deng J., Huang P., Chen H.
The extremely high level expression of human serum albumin in the
milk of transgenic mice. Transgenic Res. 2012;21:1359-1366. DOI
10.1007/s11248-012-9612-4.

Yamaji D., Kang K., Robinson G.W., Hennighausen L. Sequential activation of genetic programs in mouse mammary epithelium during
pregnancy depends on STAT5A/B concentration. Nucleic Acids Res.
2013;41:1622-1636. DOI 10.1093/nar/gks1310.

Yu Z., Meng Q., Yu H., Fan B., Yu S., Fei J., Wang L., Dai Y., Li N.
Expression and bioactivity of recombinant human lysozyme in the
milk of transgenic mice. J. Dairy Sci. 2006;89:2911-2918. DOI
10.3168/jds.S0022-0302(06)72563-2.

Yuan Y.G., An L., Yu B., Song S., Zhou F., Zhang L., Gu Y., Yu M.,
Cheng Y. Expression of recombinant human alpha-lactalbumin
in the milk of transgenic goats using a hybrid pomoter/enhancer.
J. Anal. Method. Chem. 2014;281031. DOI 10.1155/2014/281031.

Yue X.P., Zhang X.M., Wang W., Ma R.N., Deng C.J., Lan X.Y.,
Chen H., Li F., Xu X.R., Ma Y., Lei C.Z. The CSN1S1 N and F alleles identified by PCR-SSCP and their associations with milk yield
and composition in Chinese dairy goats. Mol. Biol. Rep. 2011;38:
2821-2825. DOI 10.1007/s11033-010-0428-0.

Zhou S., Cai B., He C., Wang Y., Ding Q., Liu J., Liu Y., Ding Y.,
Zhao X., Li G., Li C., Yu H., Kou Q., Niu W., Petersen B., Sonstegard T., Ma B., Chen Y., Wang X. Programmable base editing of the
sheep genome revealed no genome-wide off-target mutations. Front.
Genet. 2019;10:215. DOI 10.3389/fgene.2019.00215.

